# Identification and validation of immune and oxidative stress-related diagnostic markers for diabetic nephropathy by WGCNA and machine learning

**DOI:** 10.3389/fimmu.2023.1084531

**Published:** 2023-02-22

**Authors:** Mingming Xu, Hang Zhou, Ping Hu, Yang Pan, Shangren Wang, Li Liu, Xiaoqiang Liu

**Affiliations:** ^1^ Department of Urology, Tianjin Medical University General Hospital, Tianjin, China; ^2^ Department of Orthopedics, Tianjin Medical University General Hospital, Tianjin, China

**Keywords:** diabetic nephropathy, bioinformatic analysis, machine learning, WGCNA, biomarker

## Abstract

**Background:**

Diabetic nephropathy (DN) is the primary cause of end-stage renal disease, but existing therapeutics are limited. Therefore, novel molecular pathways that contribute to DN therapy and diagnostics are urgently needed.

**Methods:**

Based on the Gene Expression Omnibus (GEO) database and Limma R package, we identified differentially expressed genes of DN and downloaded oxidative stress-related genes based on the Genecard database. Then, immune and oxidative stress-related hub genes were screened by combined WGCNA, machine learning, and protein-protein interaction (PPI) networks and validated by external validation sets. We conducted ROC analysis to assess the diagnostic efficacy of hub genes. The correlation of hub genes with clinical characteristics was analyzed by the Nephroseq v5 database. To understand the cellular clustering of hub genes in DN, we performed single nucleus RNA sequencing through the KIT database.

**Results:**

Ultimately, we screened three hub genes, namely CD36, ITGB2, and SLC1A3, which were all up-regulated. According to ROC analysis, all three demonstrated excellent diagnostic efficacy. Correlation analysis revealed that the expression of hub genes was significantly correlated with the deterioration of renal function, and the results of single nucleus RNA sequencing showed that hub genes were mainly clustered in endothelial cells and leukocyte clusters.

**Conclusion:**

By combining three machine learning algorithms with WGCNA analysis, this research identified three hub genes that could serve as novel targets for the diagnosis and therapy of DN.

## Introduction

Diabetic nephropathy (DN), characterized by proteinuria, hypertension, and progressive reductions in kidney function, is the most common cause of end-stage renal disease in developed countries and poses a serious social and economic burden (1–3). According to studies, the number of individuals with DN is rising along with the global prevalence of diabetes, which is predicted to climb from 537 million to 783 million over the course of the next 20 years or so (4). The present course of therapy, in contrast, emphasizes renin-angiotensin system blockage, blood pressure management, and glycemic control (5). As a result, novel targets for DN diagnosis and therapy are desperately needed. With the advancement of bioinformatics, its research techniques have been actively used in recent years to explore targets for numerous illnesses, including DN.

**Figure 1:**
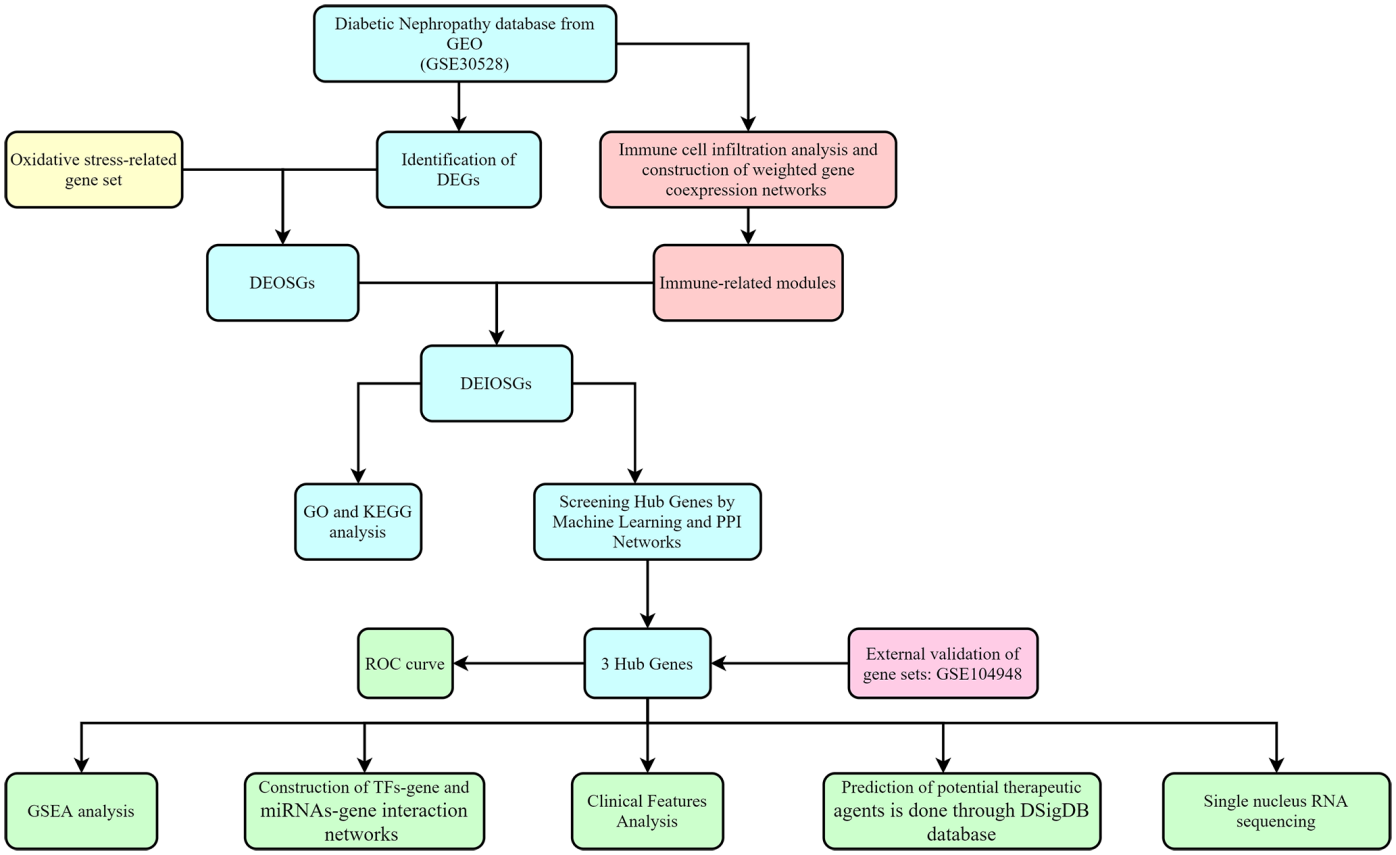
Flowchart for research.

A significant amount of data points to the importance of immune and oxidative stress in the etiology of diabetic nephropathy (6). In this research, we identified diagnostic genes for DN by a bioinformatic approach combining immune infiltration and oxidative stress and validated them with an additional external dataset, as shown in **Figure 1** for the specific study route.

## Materials and methods

### Source of data

We screened three diabetic nephropathy datasets: GSE30528 (GPL571) contained nine cases of diabetic nephropathy and thirteen controls; GSE104948 (GPL22945) served as a validation set and contained seven cases of diabetic nephropathy and eighteen controls; and GSE131882 (GPL24676) contained three early diabetic nephropathy and three control samples for single nucleus RNA sequencing. Additionally, using a relevance score of greater than 7 as a screening criterion, we were able to extract 855 genes associated with oxidative stress from the Genecard database. **Table 1** displays the pertinent details.

**Table 1:** Summary of the data sets utilized in this research and their features.

Dataset	Database	Platform	Sample
GSE30528	GEO	GPL571	9 cases of DN and 13 controls
GSE104948	GEO	GPL22945	7 cases of DN and 18 controls
Oxidative stress-related genes	Genecard	Genecard	Obtaining oxidative stress-related genes from Genecard
GSE131882	GEO	GPL24676	3 cases of DN and 3 controls

GEO, Gene Expression Omnibus; DN, Diabetic Nephropathy.

### Identification of DEGs

With |log2 fold change (FC)| > 0.5 and *p* < 0.05 as screening criteria, differentially expressed genes (DEGs) from GSE30528 were identified utilizing “Limma” R package, where log FC > 0.5, *p* < 0.05 was Up, log FC < -0.5, *p* < 0.05 was Down. The heat map and volcano map of DEG were plotted using the “Pheatmap” R package and “ggplot2” R package, respectively.

Subsequently, the obtained DEGs were intersected with 855 oxidative stress-related genes to obtain differentially expressed genes related to oxidative stress (DEOSGs).

### Immune infiltration analysis and construction of weighted gene co-expression networks

CIBERSORT employs a deconvolution algorithm to estimate the composition and abundance of immune cells in a mixture of cells based on transcriptome data. In the present study, we first assessed the proportion of 22 immune cell species in normal and diabetic nephropathy samples in GSE30528 using the CIBERSORT algorithm (7).

Weighted Gene Go-expression Network Analysis (WGCNA) is performed to identify modules of highly correlated genes, summarize the interconnections between modules and associations with external sample traits, and identify candidate biomarkers or therapeutic targets. In our research, WGCNA was constructed by the R package “WGCNA” to identify the modules with the highest relevance to immune cells in diabetic nephropathy patients (8). Specifically, we preprocessed the sample data and removed the outliers. Subsequently, the correlation matrix was constructed by the “WGCNA” software package. The optimal soft threshold was chosen to convert the correlation matrix into an adjacency matrix, and a topological overlap matrix (TOM) was created from the adjacency matrix. The TOM-based phase dissimilarity metric was utilized to categorize genes with similar expression patterns into gene modules using average linkage hierarchical clustering. The two modules with the strongest relevance to immune cells were selected as key modules for subsequent analysis.

Finally, the genes in DEOSGs and key modules were intersected, and the intersected genes were described as differentially expressed immune-related oxidative stress genes (DEIOSGs) for further study.

### Gene ontology (GO) and Kyoto Encyclopedia of Genes Genomes (KEGG) functional enrichment analysis

In this research, the “clusterProfiler” R package was implemented to conduct GO and KEGG functional enrichment analysis in R to assess gene-related biological processes (BP), molecular functions (MF), cellular components (CC), and gene-related signaling pathways.

### Screening hub genes by machine learning and PPI networks

Least Absolute Shrinkage and Selection Operator (LASSO) logistic regression analysis is a data mining method that sets the coefficients of less important variables to zero by applying the L1-penalty (lambda) in order to filter out the significant variables and construct the best classification model (9). Support Vector Machine-Recursive Feature Elimination (SVM-RFE) analysis is a supervised machine learning technique for identifying the optimal core genes by dropping the feature vectors generated by SVM (10). Random Forest (RF) analysis is a decision tree-based machine learning method that focuses on evaluating the significance of variables by scoring the importance of each variable (11). In combination with machine learning algorithms, the cytoHubba plugin is frequently applied for the identification of key genes. On the one hand, diagnostic genes from DEIOSG were assessed using the three machine learning algorithms separately (12). After that, the intersection of the three machine learning algorithms was established.

On the other hand, the STRING database was exploited to establish protein-protein interaction (PPI) networks, which Cytoscape then visualized. The differential genes were then evaluated using 12 algorithms in the cytoHubba plugin, and finally the top 10 genes for each algorithm were taken as intersection and visualized through the ImageGP platform (13).

Ultimately, the genes obtained by both methods in total were identified as hub genes.

### Clinical analysis

The Nephroseq v5 database (http://v5.nephroseq.org) (14) is a comprehensive information platform for evaluating the correlation between gene expression levels and clinical characteristics of kidney diseases. To explore the correlation between the expression of hub genes and clinical features, we mined the Nephroseq v5 database.

### GSEA analysis

We performed a single-gene GSEA analysis to investigate the possible roles of hub genes.

### Regulatory network construction and potential drug prediction

The JASPAR database (15) and the TarBase database (16) were accessed by the NetworkAnalyst (https://www.networkanalyst.ca/) (17) to predict transcription factors (TFs) and miRNAs, respectively. Subsequently, the results were visualized using Cytoscape software.

We used the Enrichr platform (https://amp.pharm.mssm.edu/Enrichr/) (18) to access the DSigDB database (19) for potential drug prediction.

### Single nucleus RNA sequencing

A single-cell sequencing database for kidney disease called the Kidney Integrative Transcriptomics (K.I.T.) database was developed by Ben Humphrey’s lab at Washington University (http://humphreyslab.com/SingleCell/) (20). To explore the distribution of hub genes in cell groups, we applied the database for analysis and visualization of the results. In one of them, we used single nucleus RNA sequencing data from diabetic nephropathy that was initially taken from the GSE131882 dataset.

### Statistical analysis

GraphPad Prism 8.0 (GraphPad Software, CA, USA) was implemented to conduct the statistical analysis. The diagnostic value of hub genes was evaluated with ROC curve analysis. Hub genes were analyzed for correlation with clinical features *via* Pearson analysis. An unpaired t-test was performed for the assessment of hub gene differential expression. *P* < 0.05 was defined as statistically significant.

## Results

### Identification of DEGs

**Figure 2:**
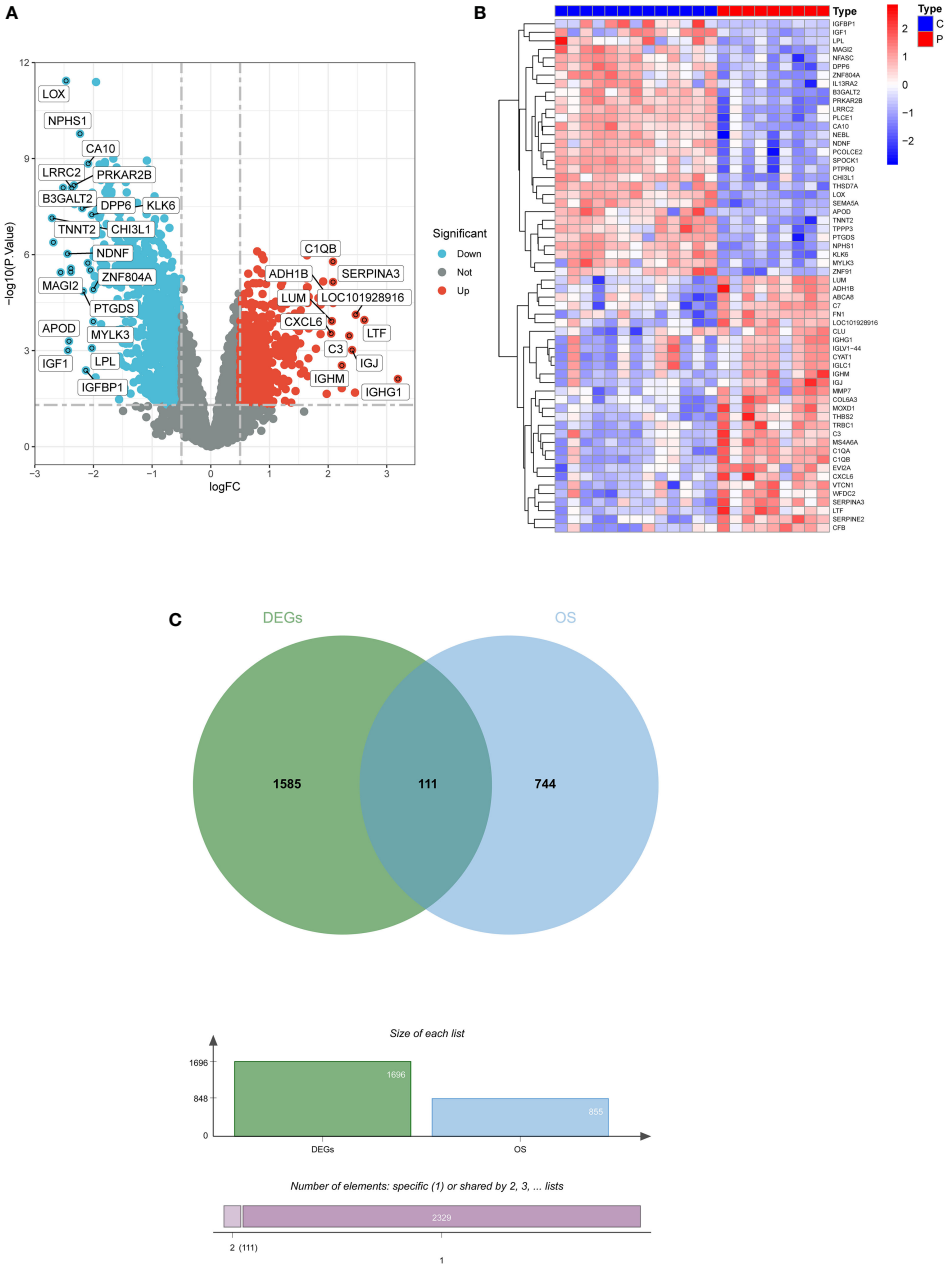
Screening for DEGs. **(A)** Volcano plot of DEGs in GSE30528. **(B)** Heatmap of DEGs in GSE30528. **(C)** Venn diagrams of DEOSGs. DEGs, differentially expressed genes; DEOSGs, differentially expressed genes related to oxidative stress.

A total of 1696 DEGs were acquired from GSE30528, and another 855 oxidative stress-related genes were mined from the Genecard database, and 111 DEOSGs were generated by taking the intersection of the two (**Figures 2A–C**).

### Immune infiltration analysis and construction of weighted gene coexpression networks

**Figure 3:**
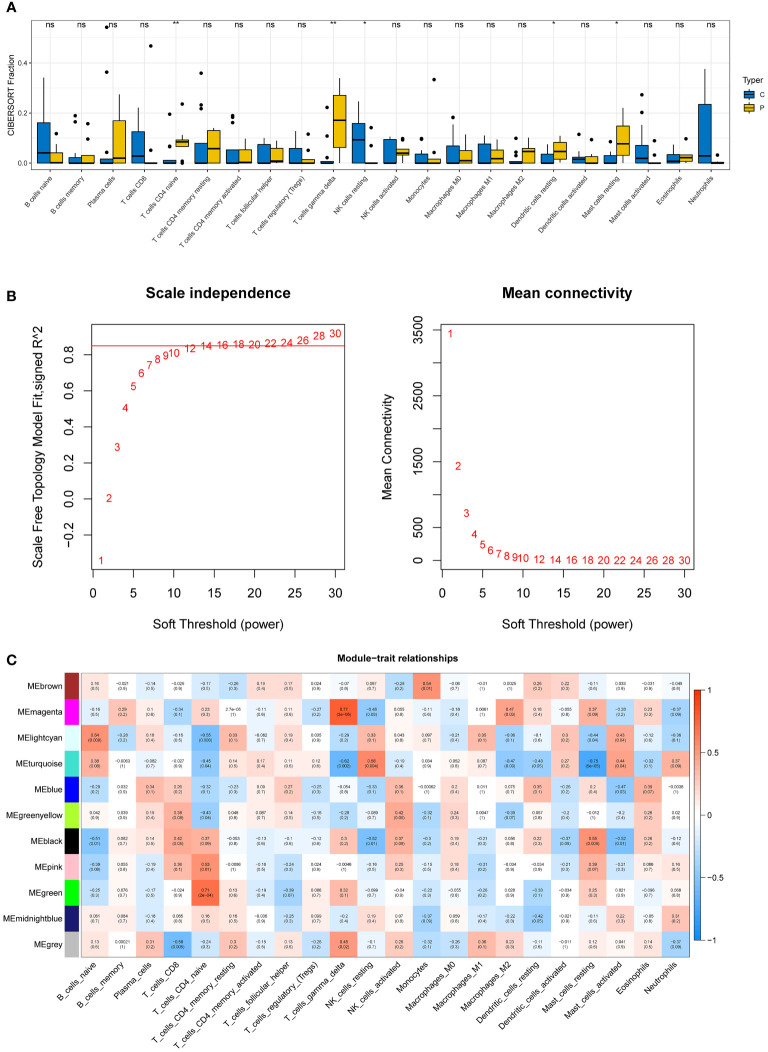
Immune infiltration analysis and construction of weighted gene co-expression networks. **(A)** 22 immune cells in samples with normal and diabetic nephropathy in GSE30528. **(B)** Choosing the best soft-threshold power. **(C)** 11 modules revealed by the WGCNA. WGCNA, weighted gene co-expression network analysis.

Five immune cell types, including T cells CD4 naive, T cells gamma delta, NK cells resting, Dendritic cells resting, and mast cells resting, were demonstrated to be comparable between DN and control samples using the CIBERSORT algorithm (**Figure 3A**).

The soft-threshold power in this research was calibrated to 14 (scale-free *R^2^* = 0.85) (**Figure 3B**). Last but not least, a sum of 11 modules was revealed by the WGCNA analysis (**Figure 3C**). In particular, the green module and the magenta module had strong positive correlations with T cell CD4 naive and gamma delta subsets, respectively. Due to their significance in association with immunological infiltrating cells, the green and magenta modules were considered for additional investigation.

### Acquisition and functional enrichment analysis of DEIOSGs

**Figure 4:**
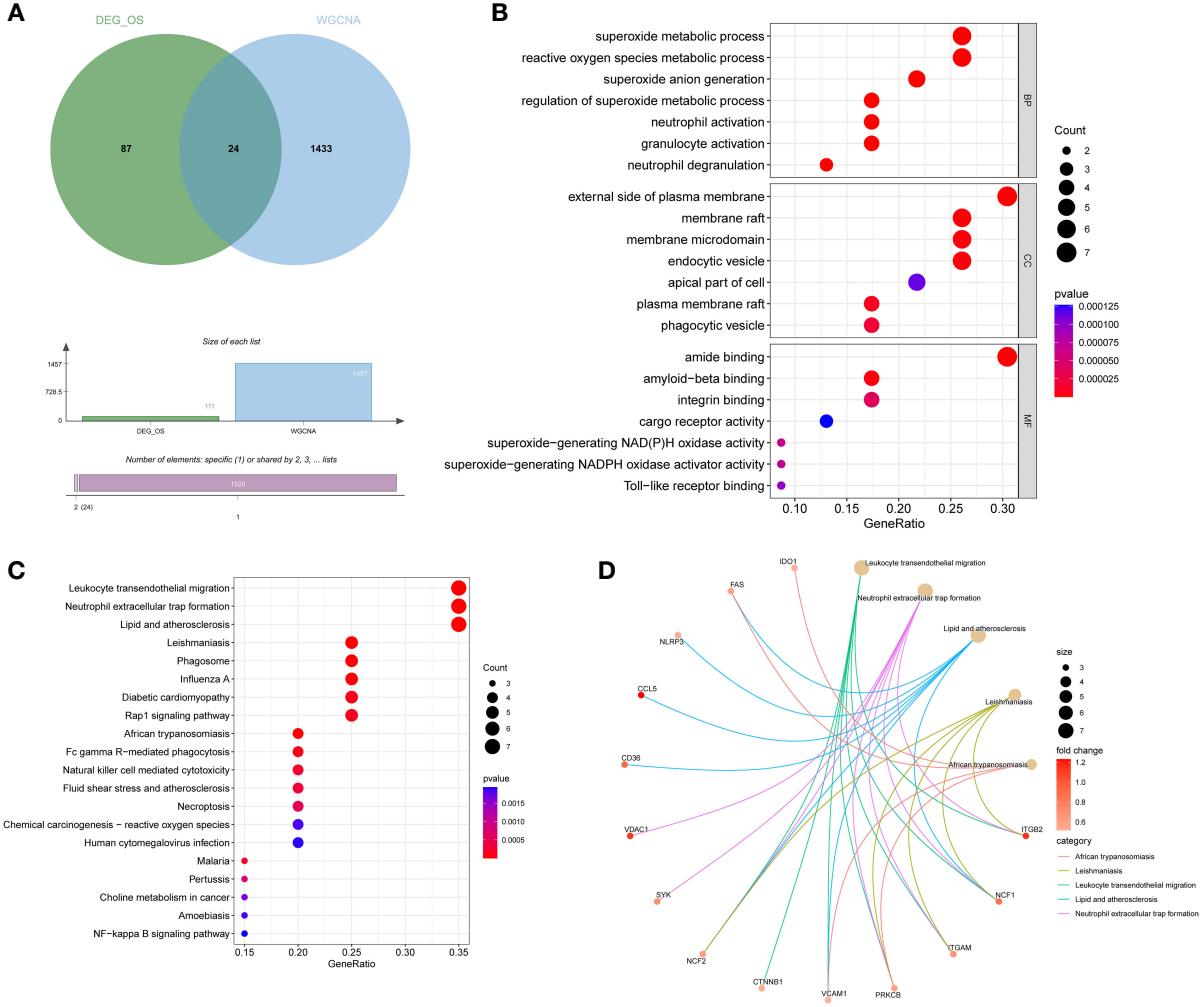
Acquisition and functional enrichment analysis of DEIOSGs. **(A)** Venn diagrams of DEIOSGs. **(B)** The GO outcomes are displayed with a bubble plot. **(C)** A bubble plot was constructed to illustrate the KEGG outcomes. **(D)** Results of KEGG are depicted on circle charts. DEIOSGs, differentially expressed immune-related oxidative stress genes; GO, Gene Ontology; KEGG, Kyoto Encyclopedia of Genes and Genomes; BP, biological process; CC, cellular component; MF, molecular function.

DEIOSGs are the genes that overlap DEOSGs with the magenta and green modules generated by WGCNA, and a total of 24 DEIOSGs were identified (**Figure 4A**).

Furthermore, we performed the functional enrichment of 24 DEIOSGs *via* GO and KEGG. In the BP assessment, DEIOSGs were mostly engaged in superoxide metabolic processes, neutrophil activation, and other functions. DEIOSGs have been localized to the external side of the plasma membrane, endocytic vesicle, and other structures in CC. DEIOSG changes associated with MF include amide binding, integrin binding, and superoxide-generating NAD(P)H oxidase activity (**Figure 4B**). According to KEGG analysis, DEIOSGs are particularly abundant in leukocyte transendothelial migration, neutrophil extracellular trap formation, lipid and atherosclerosis, diabetic cardiomyopathy, natural killer cell mediated cytotoxicity and other pathways (**Figures 4C, D**).

### Screening hub genes by machine learning and PPI networks

**Figure 5:**
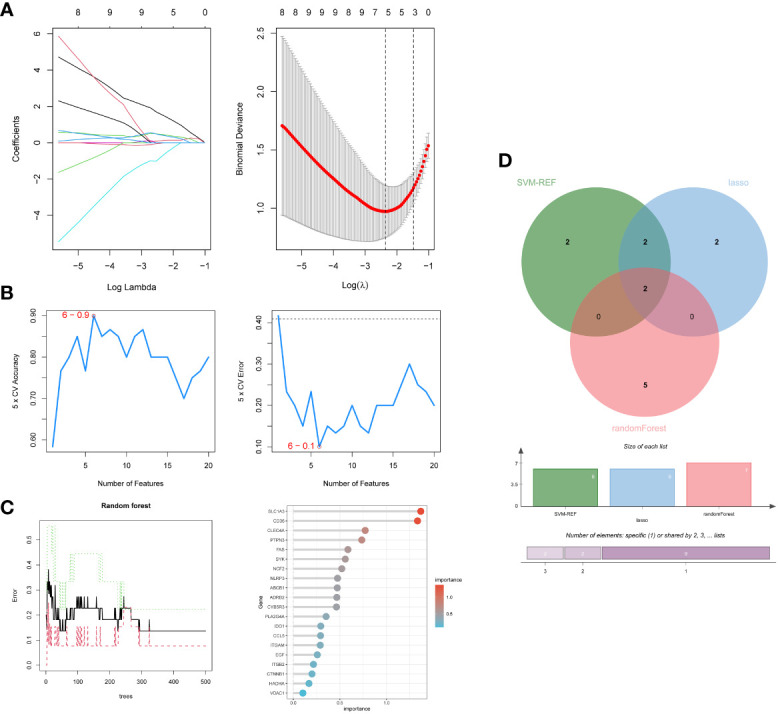
Screening hub genes by machine learning. **(A)** LASSO regression algorithm. **(B)** SVM-RFE algorithm. **(C)** RF algorithm. **(D)** Venn diagrams for three algorithms. LASSO, Least Absolute Shrinkage and Selection Operator; SVM-RFE, Support Vector Machine-Recursive Feature Elimination; RF, Random Forest.

**Figure 6:**
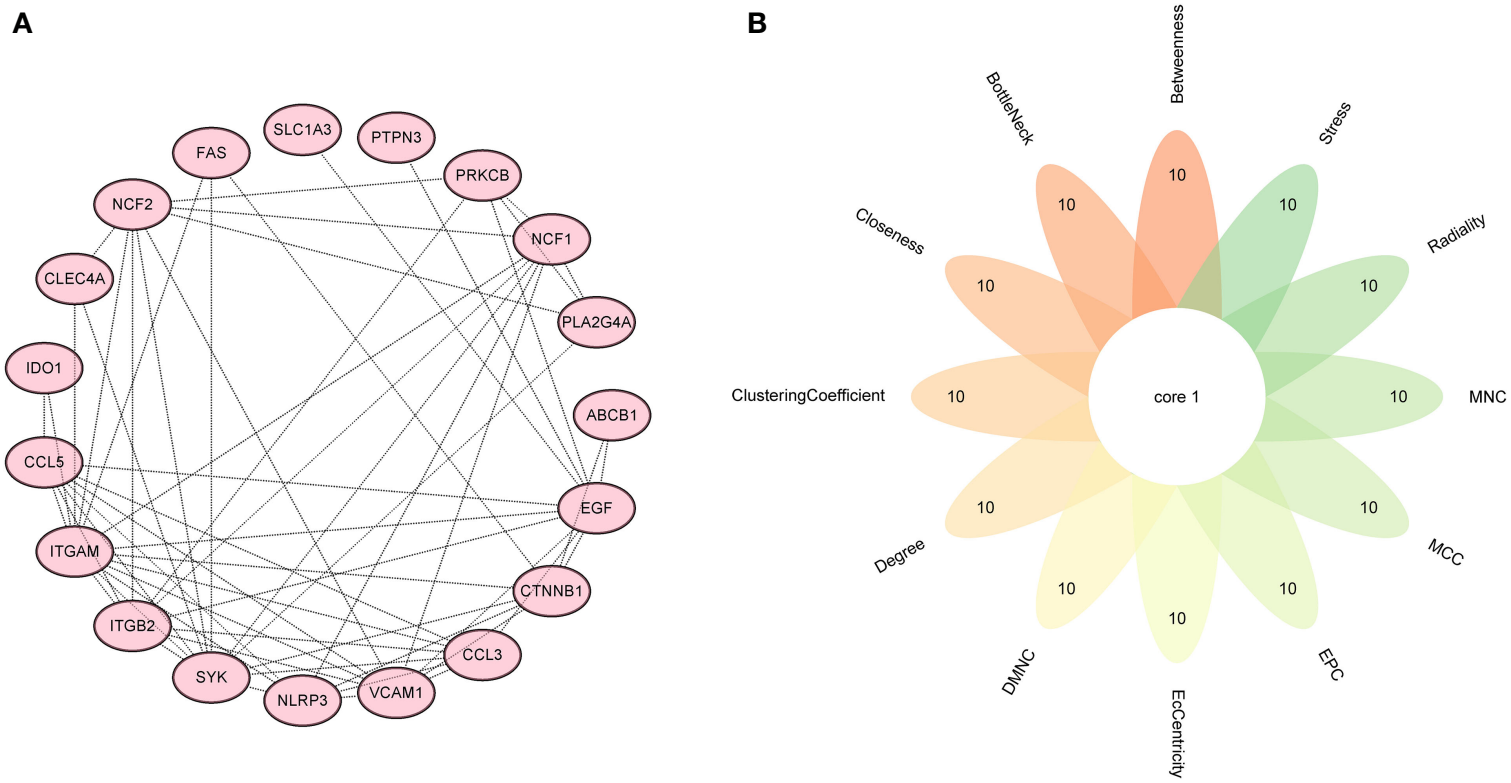
Screening hub genes by PPI network. **(A)** PPI network. **(B)** Venn diagrams for 12 algorithms in cytoHubba plugin. PPI, protein-protein interaction.

Firstly, 6 genes were extracted from DEIOSGs using the LASSO regression algorithm (**Figure 5A**). Secondly, the SVM-RFE algorithm identified 6 genes (**Figure 5B**). Then, 7 genes were selected by the RF algorithm (**Figure 5C**). Subsequently, the three were overlapped by the Venn diagram and finally two genes were obtained, namely CD36 and SLC1A3 (**Figure 5D**). Meanwhile, from the PPI network, we obtained a gene, namely ITGB2, through the cytoHubba plugin (**Figures 6A, B**). Finally, a total of 3 hub genes were identified by both methods, all of which were up-regulated.

### Expression of hub genes and validation of external datasets

**Figure 7:**
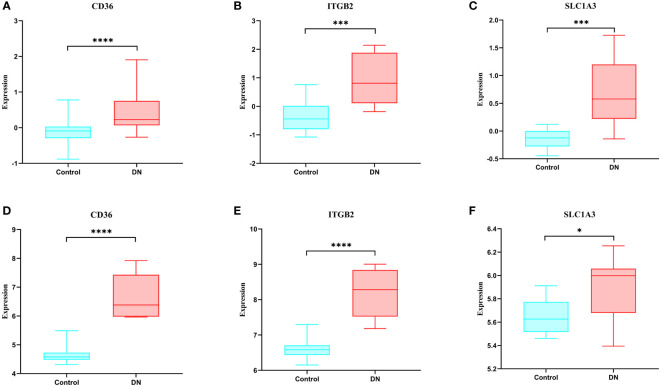
Expression of hub genes and validation of external datasets. **(A-C)** Expression of hub genes in the GSE30528 dataset. **(D-F)** Expression of hub genes in the GSE104948 dataset. * p<0.05; ** p<0.01; *** p<0.001; **** p<0.0001.

When compared to the normal control sample, we discovered in the GSE30528 dataset that these genes were expressed more highly in DN (**Figures 7A–C**). We next confirmed the expression of these genes using another dataset, and the results revealed that these genes were likewise more strongly expressed in DN than control in GSE104948, and they were all statistically significant (**Figures 7D–F**).

### ROC curve analysis

**Figure 8:**
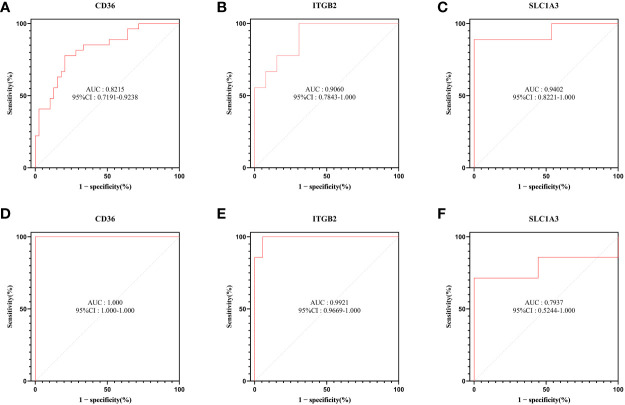
ROC curve analysis. **(A–C)** Hub genes in the GSE30528 dataset were analyzed using ROC curves. **(D–F)** Hub genes in the GSE104948 dataset were analyzed using ROC curves.

To explore the diagnostic efficacy of the 3 hub genes, we implemented a ROC curve analysis in which hub genes with an AUC value > 0.7 were used as diagnostic markers. In the GSE30528 dataset, the AUC values were 0.8215 for CD36, 0.9402 for SLC1A3, and 0.9060 for ITGB2 (**Figures 8A–C**).

In the GSE104948 dataset, the AUC values of CD36 were 1.000 (95% CI: 1.000-1.000), AUC values of SLC1A3 were 0.7937 (95% CI: 0.5244-1.000), AUC values of ITGB2 were 0.9921 (95% CI: 0.9669-1.000) (**Figures 8D–F**).

### GSEA analysis

**Figure 9:**
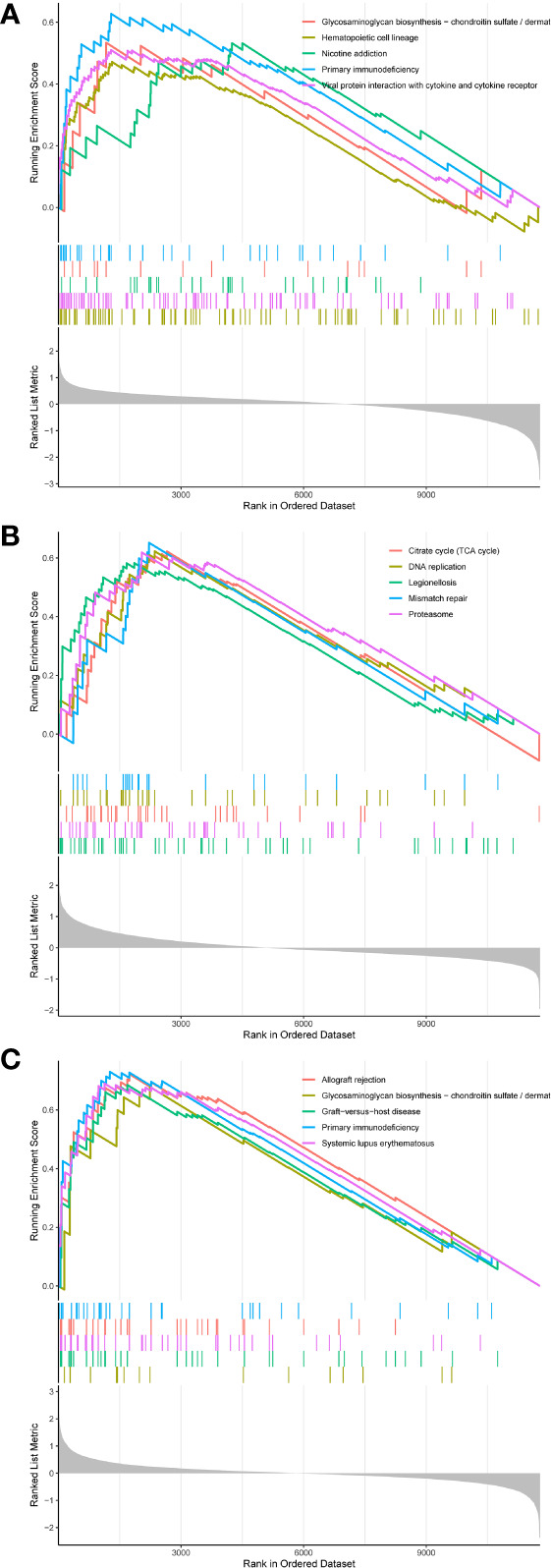
**(A-C)** GSEA analysis of hub genes.

According to GSEA findings, the CD36 high expression group was highly enriched for primary immunodeficiency and viral protein interaction with cytokines and cytokine receptors (**Figure 9A**). The ITGB2 high expression group was mostly concentrated in the citrate cycle (TCA cycle) and proteasome (**Figure 9B**). Allograft rejection, primary immunodeficiency, and systemic lupus erythematosuswere all associated with increased SLC1A3 expression (**Figure 9C**).

### Clinical analysis

**Figure 10:**
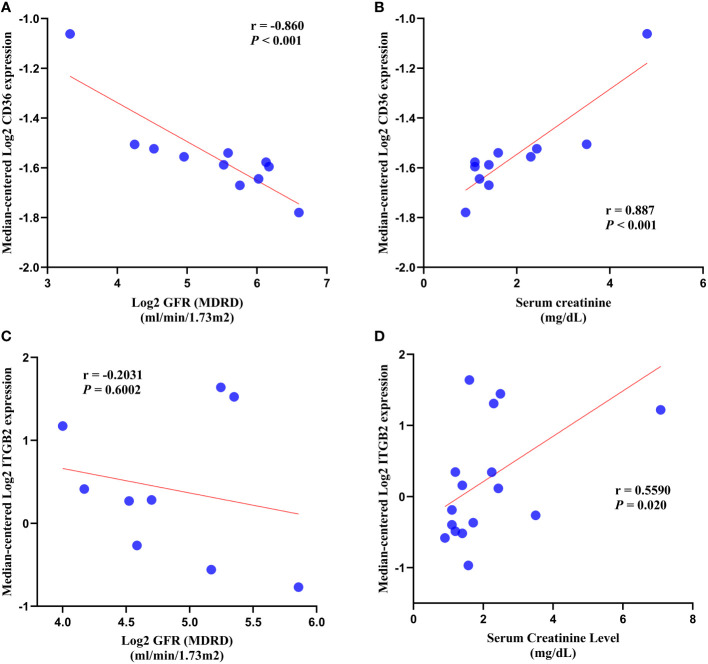
Correlation analysis**. (A, B)** Correlation analysis of CD36 with GFR and serum creatinine. **(C, D)** Correlation analysis of ITGB2 with GFR and serum creatinine. GFR, glomerular filtration rate.

In DN patients, correlation analysis revealed a negative correlation between CD36 expression and glomerular filtration rate (GFR) (*r* = -0.860, *p* < 0.001) and a positive correlation between CD36 expression and serum creatinine (*r* = 0.887, *p* < 0.001) (**Figures 10A, B**). ITGB2 expression was negatively correlated with glomerular filtration rate (GFR) (*r* = -0.2031, *p* = 0.6002) but not statistically different, whereas ITGB2 expression was positively correlated with serum creatinine (*r* = 0.5590, *p* = 0.020) (**Figures 10C, D**).

### Regulatory network construction and potential drug prediction

**Figure 11:**
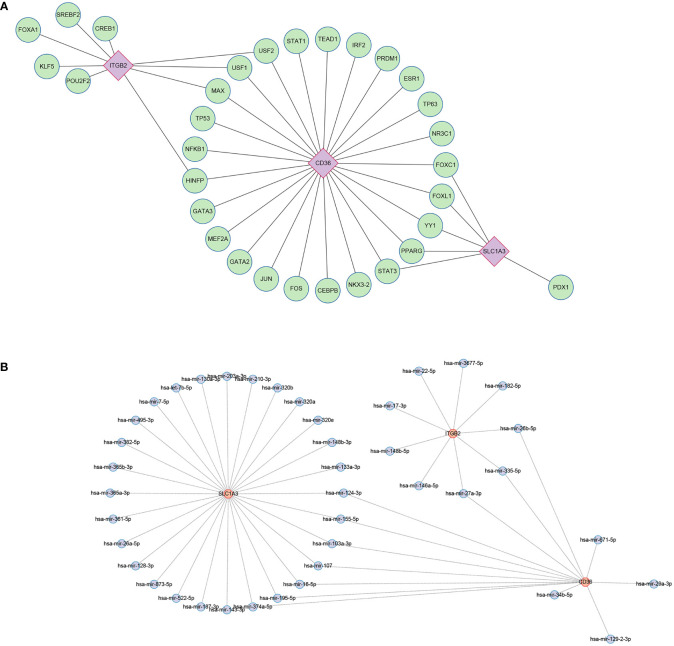
Regulatory network. **(A)** Interaction network of TFs and genes for the hub genes. **(B)** Network of interactions between miRNAs and the hub genes. TF, transcription factors; miRNA, microRNA.

Using the JASPAR database, 31 TFs were finally obtained, among which, there were 9 TFs with degree≥2, and they were FOXC1, FOXL1, YY1, PPARG, STAT3, HINFP, MAX, USF1, USF2 (**Figure 11A**). Possible miRNAs were predicted by the TarBase database with 10 miRNAs of degree≥2 (**Figure 11B**).

Eighty-seven potential therapeutic agents were screened in the DSigDB database with a cut-off value of Adjusted p-value < 0.05 (**Supplementary Table 1**).

### Single nucleus RNA sequencing

**Figure 12:**
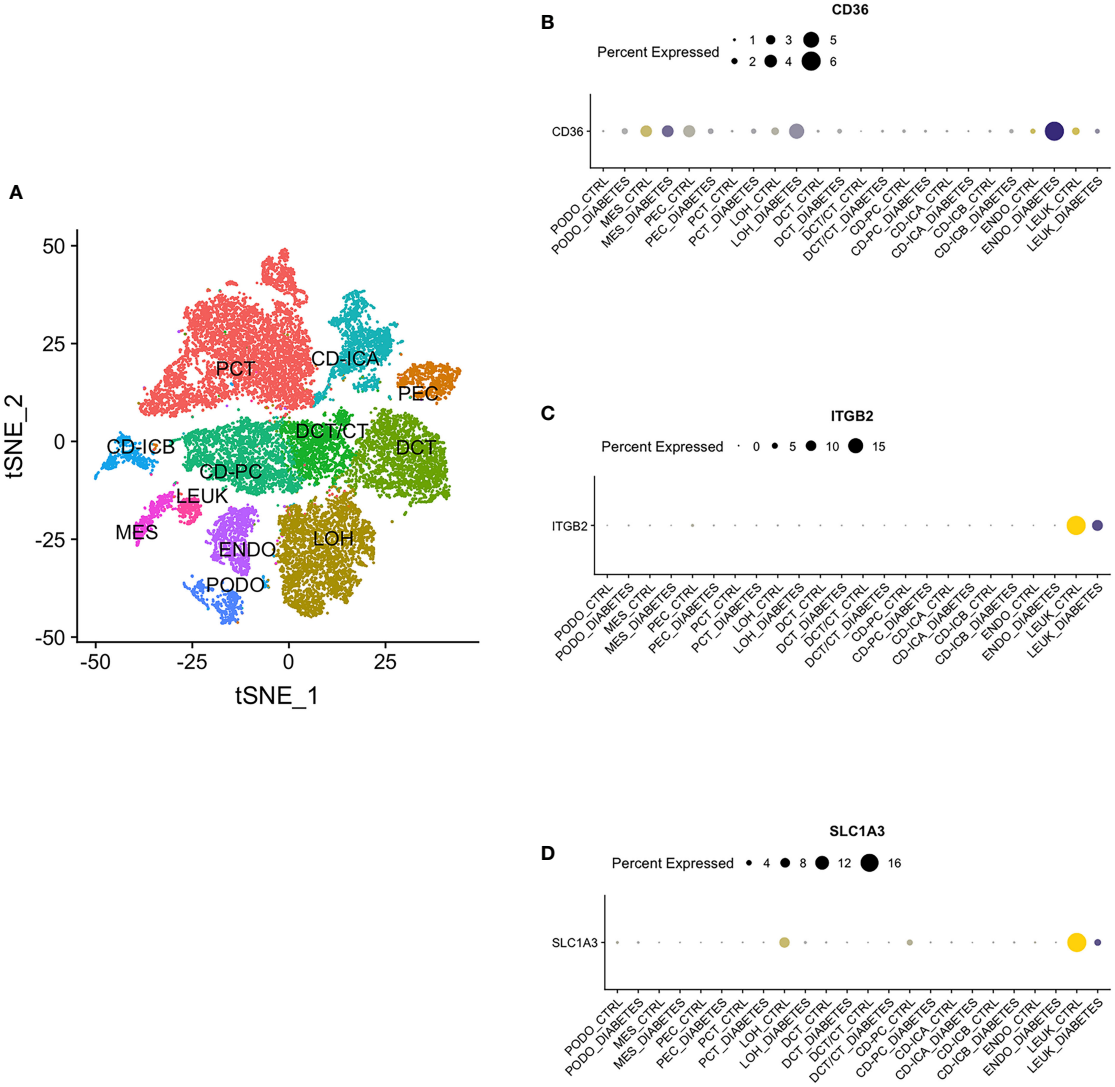
Single Nucleus RNA Sequencing. **(A)** The distribution of hub genes in 12 cell groups. **(B)** CD36. **(C)** ITGB2. **(D)** SLC1A3. PCT, proximal convoluted tubule; CD, collecting duct; ICA, Type A intercalated cells; ICB, Type B intercalated cells; PEC, parietal epithelial cells; PC, principal cell; DCT, distal convoluted tubule; CT, connecting tubule; LOH, loop of Henle; PODO, podocyte; ENDO, endothelium; MES, mesangial cell; LEUK, leukocyte.

By single nucleus RNA sequencing, we determined the distribution of CD36, ITGB2 and SLC1A3 in 12 cell groups (**Figure 12A**), among which CD36 was mainly distributed in endothelium and ITGB2 and SLC1A3 were highly expressed in leukocyte (**Figures 12B-D**).

## Discussion

Diabetic nephropathy is triggered by a combination of several factors (21). However, its specific mechanisms remain to be explored. Due to the heterogeneity of individuals, the present therapeutic effects for diabetic nephropathy are constrained, making the necessity for novel molecular pathways that contribute to DN therapy and diagnosis essential. The progression of DN has been determined to be significantly controlled by immune infiltration and oxidative stress (22, 23). Meanwhile, with the progression of a diverse range of informatics technologies, machine learning algorithms and WGCNA have become more mature and are widely applied for the prediction of disease markers and therapeutic targets. In this research, we retrieved transcriptomic datasets from the GEO database and, combining machine learning, WGCNA, and PPI networks, identified a set of three immune and oxidative stress-related hub genes, namely CD36, ITGB2, and SLC1A3, and validated them with an additional dataset. We implemented ROC curve analysis to assess the diagnostic value of hub genes, and the results showed that all three hub genes had excellent diagnostic efficacy.

CD36, commonly regarded as a scavenger receptor, is located in a wide range of renal cells (24), which is consistent with our single nucleus RNA sequencing analysis. Lipid metabolism, immunological inflammation, and renal fibrosis are its key areas of involvement. According to research, a possible therapeutic target for the prevention of renal fibrosis may be CD36 (25). Little research has been performed on the function of CD36 in immune-related oxidative stress, even though CD36 is broadly investigated in the pathogenesis of DN. In this research, we discovered that CD36 expression was elevated in the renal tissues of individuals with diabetic nephropathy and had a diagnostic accuracy value (AUC > 0.80). Cohort studies revealed that sCD36 levels in plasma and urine were raised in DN patients and correlated with DN severity, indicating that sCD36 may be a diagnostic marker for DN progression (26). Furthermore, the mechanism of CD36 engagement in DN is mostly attributed to oxidative stress triggered by lipid deposition (27), which is consistent with the results of our functional enrichment analysis. Hou Y. et al. revealed that CD36 contributed to DN progression by triggering epithelial-mesenchymal transition (EMT) through the induction of reactive oxygen species (ROS) production (28). Additionally, the outcomes of animal studies suggested that inhibiting CD36 might shield diabetic mice from kidney harm and oxidative stress (29).

ITGB2, a member of the integrin family, is mostly expressed in immune cells and is connected to a variety of metabolic pathways as well as immune functions such as leukocyte extravasation (30). Similarly, ITGB2 is crucial for the growth of tumors. For instance, it is primarily in charge of the invasion and metastasis of tumor cells in gliomas, which is closely connected to the immune microenvironment (31). The engagement of ITGB2 in DN development, however, has received relatively little research. In our research, we observed that ITGB2 with upregulated expression also has excellent diagnostic efficacy (AUC > 0.90). Based on the most recent experimental research, ITGB2 is essential for the progression of diabetes, and the ITGB2 gene deficiency may hopefully prevent the disease (32). This paves the way for ITGB2 to become a diagnostic marker for DN. Furthermore, there is a growing consensus that EMT is essential for the development of DN (33, 34). And ITGB2 is also closely related to the regulation of EMT (35, 36).

SLC1A3, an aspartate and glutamate transporter, is abundantly expressed in cerebral and tumor tissues and is associated with immune inflammation as well as proliferation and metastasis of tumors (37). It has also been proposed that SLC1A3 is involved in the amino acid-related metabolism of adipocytes (38). Furthermore, insulin has been demonstrated to regulate the expression and activity of SLC1A3 (39). And SLC1A3 is mainly involved in diabetic retinopathy in diabetic complications (40). In our results, SLC1A3 is expressed more strongly in DN patients than in healthy controls.

According to the results of our investigation, CD36, which was upregulated in renal tissue, was significantly linked to reduced GFR and increased serum creatinine, implying that CD36 expression may be associated with reduced renal function in patients with DN. ITGB also has a similar presentation.

As we all know, the two key mechanisms in the progression of DN are oxidative stress and immunity, and they are inexorably intertwined. Hyperglycemia is a central factor in kidney damage in DN patients (41). On one hand, hyperglycemia induces oxidative stress by activating the renin-angiotensin-aldosterone system (RAAS), which leads to renal injury (42). On the other hand, the stress caused by persistent hyperglycemia can lead to a high production of inflammatory molecules and the accumulation of immune complexes, a process that is closely related to immune cells such as mast cells (43). The results of immune infiltration analysis also suggest that mast cells, NK cells and T cells are closely associated with the development of DN. In addition, the results of our functional enrichment analysis also suggest that DEIOSGs are mainly enriched in immune and oxidative stress-related pathways. Therefore, therapeutic strategies targeting immune and oxidative stress are particularly important and promising.

However, this study has several limitations. The evidence is based on publicly available data, and although we performed expression validation with another dataset, further experiments are needed to validate these 3 diagnostic markers before they can be applied to the clinic.

In conclusion, by combining three machine learning algorithms with WGCNA analysis, this research identified three hub genes that could serve as novel targets for the diagnosis and therapy of DN.

## Data availability statement

The datasets presented in this study can be found in online repositories. The names of the repository/repositories and accession number(s) can be found in the article/**Supplementary Material**.

## Author contributions

The manuscript was conceived by MX. PH and MX were responsible for software operation and analysis. PH, HZ, and YP performed the data compilation. Data analysis and interpretation were performed by HZ and SW. MX completed the manuscript. XL and LL were responsible for manuscript review and revision. The article was submitted with the authorization of all authors who also contributed to the article.
