# Supplementary material for: Identification and validation of immune and oxidative stress-related diagnostic markers for diabetic nephropathy by WGCNA and machine learning
Source: Front Immunol. 2023 Feb 22;14:1084531. doi: 10.3389/fimmu.2023.1084531 (PMC9992203; doi:10.3389/fimmu.2023.1084531)
Supplement: Supplementary file 1 [file Table_1.docx]

**Supplementary Table 1.** Prediction of candidate drugs for hub genes.

| Name of drugs | P-value | Adjusted p-value | Gene |
| --- | --- | --- | --- |
| ACMC-20mvek CTD 00002629 | 0.0000755 | 0.009438 | ITGB2; CD36 |
| Alitretinoin CTD 00003402 | 0.0003363 | 0.009631 | ITGB2; CD36 |
| Bisphenol A CTD 00000312 | 0.0002513 | 0.009631 | ITGB2; CD36; SLC1A3 |
| LY 294002 CTD 00003061 | 0.0003853 | 0.009631 | ITGB2; CD36 |
| wortmannin CTD 00000504 | 0.0001602 | 0.009631 | ITGB2; CD36 |
| aspirin CTD 00005447 | 0.002321 | 0.02231 | ITGB2; CD36 |
| Ginsenoside Rh1 CTD 00003920 | 0.001649 | 0.02231 | ITGB2 |
| Malondialdehyde CTD 00006237 | 0.002099 | 0.02231 | CD36 |
| Phorbol 12-myristate 13-acetate CTD 00006852 | 0.001725 | 0.02231 | ITGB2; CD36 |
| Rifaximin CTD 00001936 | 0.002248 | 0.02231 | CD36 |
| Ropivacaina [Spanish] CTD 00001768 | 0.002099 | 0.02231 | ITGB2 |
| Tamibarotene CTD 00002527 | 0.002304 | 0.02231 | ITGB2; CD36 |
| theophylline CTD 00006862 | 0.001648 | 0.02231 | ITGB2; CD36 |
| candesartan CTD 00002974 | 0.002698 | 0.02409 | CD36 |
| Anandamide CTD 00002912 | 0.003147 | 0.02424 | CD36 |
| CLODRONATE DISODIUM BOSS | 0.003296 | 0.02424 | ITGB2 |
| losartan CTD 00007310 | 0.003147 | 0.02424 | CD36 |
| AFLATOXIN B1 CTD 00007128 | 0.003656 | 0.02539 | ITGB2; CD36; SLC1A3 |
| Acteoside CTD 00002463 | 0.006436 | 0.02595 | ITGB2 |
| cacodylic acid CTD 00005554 | 0.004942 | 0.02595 | CD36 |
| cerivastatin CTD 00003073 | 0.004344 | 0.02595 | ITGB2 |
| CHEMBL35349 CTD 00001274 | 0.006287 | 0.02595 | ITGB2 |
| GADOLINIUM BOSS | 0.006436 | 0.02595 | ITGB2 |
| lidocaine CTD 00006207 | 0.004493 | 0.02595 | ITGB2 |
| N-Formyl-Met-Leu-Phe BOSS | 0.006287 | 0.02595 | ITGB2 |
| N-formylmethionylleucylphenylalanine CTD 00006360 | 0.00539 | 0.02595 | ITGB2 |
| oxozinc CTD 00007012 | 0.00539 | 0.02595 | CD36 |
| pentoxifylline CTD 00006487 | 0.005988 | 0.02595 | ITGB2 |
| Telmisartan CTD 00003021 | 0.006436 | 0.02595 | CD36 |
| Tetradioxin BOSS | 0.005839 | 0.02595 | ITGB2 |
| yc-1 CTD 00003160 | 0.005092 | 0.02595 | CD36 |
| 139890-68-9 CTD 00002746 | 0.006884 | 0.02608 | ITGB2 |
| Acetovanillone CTD 00002374 | 0.006735 | 0.02608 | CD36 |
| methacholine BOSS | 0.007482 | 0.02751 | ITGB2 |
| choline CTD 00005662 | 0.00778 | 0.02779 | CD36 |
| dl-Thioctic acid CTD 00006215 | 0.008228 | 0.0278 | CD36 |
| Methoprene BOSS | 0.008228 | 0.0278 | ITGB2 |
| GW9662 CTD 00004071 | 0.008824 | 0.02876 | CD36 |
| sulfasalazine CTD 00006719 | 0.008973 | 0.02876 | ITGB2 |
| Electrocorundum CTD 00005364 | 0.009272 | 0.02897 | CD36 |
| bisindolylmaleimide i CTD 00002749 | 0.00957 | 0.02918 | ITGB2 |
| estradiol CTD 00005920 | 0.01019 | 0.03033 | ITGB2; CD36; SLC1A3 |
| benzo[a]pyrene CTD 00005488 | 0.01082 | 0.03147 | ITGB2; CD36; SLC1A3 |
| 9,10-Phenanthrenequinone TTD 00001360 | 0.01374 | 0.03857 | ITGB2 |
| bezafibrate CTD 00005506 | 0.01389 | 0.03857 | CD36 |
| Calcimycin CTD 00005287 | 0.01493 | 0.0397 | ITGB2 |
| Toxoflavin TTD 00011503 | 0.01493 | 0.0397 | ITGB2 |
| 9,10-Phenanthrenequinone CTD 00000222 | 0.01611 | 0.04028 | ITGB2 |
| cholecalciferol CTD 00005655 | 0.01567 | 0.04028 | ITGB2 |
| nitric oxide CTD 00006402 | 0.01582 | 0.04028 | ITGB2 |
| Pioglitazone CTD 00002515 | 0.01671 | 0.04095 | CD36 |
| resveratrol CTD 00002483 | 0.01821 | 0.04378 | ITGB2; CD36 |
| L-histidine BOSS | 0.01908 | 0.045 | ITGB2 |
| 4-aminobutyric acid BOSS | 0.02676 | 0.04503 | SLC1A3 |
| 9001-31-4 BOSS | 0.02011 | 0.04503 | ITGB2 |
| AGN-PC-0JHFVD BOSS | 0.02838 | 0.04503 | ITGB2 |
| cerivastatin BOSS | 0.02867 | 0.04503 | ITGB2 |
| diphenylpyraline BOSS | 0.02499 | 0.04503 | ITGB2 |
| ethisterone BOSS | 0.02867 | 0.04503 | ITGB2 |
| eugenol CTD 00005949 | 0.0241 | 0.04503 | CD36 |
| furosemide BOSS | 0.02882 | 0.04503 | ITGB2 |
| HEXACHLOROETHANE BOSS | 0.02823 | 0.04503 | ITGB2 |
| hydroquinone CTD 00001535 | 0.02573 | 0.04503 | ITGB2 |
| ibuprofen CTD 00006137 | 0.02233 | 0.04503 | CD36 |
| Insulin BOSS | 0.02838 | 0.04503 | ITGB2 |
| IRON CTD 00006166 | 0.02691 | 0.04503 | ITGB2 |
| Isoguanine BOSS | 0.02853 | 0.04503 | ITGB2 |
| L-aspartic acid BOSS | 0.02573 | 0.04503 | SLC1A3 |
| L-glutamic acid BOSS | 0.02823 | 0.04503 | SLC1A3 |
| L-glutamine BOSS | 0.02823 | 0.04503 | SLC1A3 |
| midecamycin HL60 UP | 0.02617 | 0.04503 | CD36 |
| Oxazolone CTD 00006449 | 0.01952 | 0.04503 | CD36 |
| Phetharbital BOSS | 0.02853 | 0.04503 | ITGB2 |
| progesterone CTD 00006624 | 0.02576 | 0.04503 | ITGB2; SLC1A3 |
| Simvastatin and niacin BOSS | 0.02867 | 0.04503 | ITGB2 |
| staurosporine CTD 00007273 | 0.02543 | 0.04503 | ITGB2 |
| Superoxide BOSS | 0.02808 | 0.04503 | ITGB2 |
| TITANIUM DIOXIDE CTD 00000489 | 0.02174 | 0.04503 | CD36 |
| trimethoprim BOSS | 0.02838 | 0.04503 | ITGB2 |
| VANADIUM CTD 00006979 | 0.02233 | 0.04503 | CD36 |
| 2,2',4,4',5,5'-Hexachlorobiphenyl CTD 00000731 | 0.03102 | 0.04562 | SLC1A3 |
| Capsaicin BOSS | 0.03 | 0.04562 | SLC1A3 |
| dimethyl sulfoxide CTD 00005842 | 0.03088 | 0.04562 | ITGB2 |
| N-methyl-D-aspartic acid BOSS | 0.03014 | 0.04562 | SLC1A3 |
| Sodium dichromate CTD 00000827 | 0.03102 | 0.04562 | CD36 |
| Cube root extract CTD 00006707 | 0.03147 | 0.04573 | CD36 |
| Retinoic acid BOSS | 0.0344 | 0.04942 | ITGB2 |
